# Chocolate odor enhances resistance exercise performance through appetite suppression in the fasted state: an exploratory study

**DOI:** 10.3389/fphys.2026.1834757

**Published:** 2026-07-09

**Authors:** Xiaohan Fan, Hengzhi Deng, Jia Yang Ng, Ahmad Amirul Hazim bin Ab Aziz, Mohamed Nashrudin bin Naharudin

**Affiliations:** Faculty of Sports and Exercise Science, University of Malaya, Kuala Lumpur, Malaysia

**Keywords:** Appetite regulation, cephalic-phase, chocolate odor, olfaction, resistance exercise

## Abstract

**Introduction:**

Olfactory stimuli may alter appetite, affective responses, and exercise performance through psychobiological processes. However, whether odor-induced changes in appetite and pleasantness translate into measurable improvements in resistance exercise performance remains unclear. This exploratory study investigated whether exposure to chocolate-related odors alters appetite perception, odor pleasantness, and resistance exercise capacity in moderately trained men.

**Methods:**

Twenty-three participants completed a randomized, double-blind, crossover trial with three odor conditions presented as freshly prepared liquid stimuli: 90% dark chocolate, 60% milk chocolate, and a water-based control. Appetite perception and odor pleasantness were assessed using 100-mm visual analog scales, and perceived exertion was assessed using the Borg CR10 scale. Ratings were obtained at baseline and after odor exposure. Participants then performed repeated 10-repetition leg-extension sets at 80% of 10RM to volitional exhaustion with 3.5-min rest intervals. Odors were presented before each set, followed by assessments of appetite, pleasantness, and ratings of perceived exertion.

**Results:**

Condition significantly affected total repetitions (Wald *χ*²(2) = 71.15, *p* < 0.01) and total sets (Wald *χ*²(2) = 21.27, *p* < 0.01). Total repetitions were greater with 90% dark chocolate than with control (+18, 95% CI 13.8 to 22.1, *p* < 0.01) and greater with 60% milk chocolate than with control (+9, *p* < 0.01). Ratings of perceived exertion increased across sets (*p* < 0.01) but did not differ between conditions. Before exercise, 90% dark chocolate reduced appetite-related ratings, whereas 60% milk chocolate increased odor pleasantness.

**Discussion:**

These findings indicate that chocolate-related odors can modulate appetite and hedonic responses while increasing resistance-exercise volume, without clear increases in post-set perceived exertion. Chocolate-related odors may offer a non-nutritive approach to support fasted resistance exercise, although further work is needed to confirm the mechanisms and generalizability of these effects.

## Highlights

- Chocolate odor exposure acutely enhanced resistance exercise volume without increasing perceived exertion.
- Dark chocolate odor predominantly suppressed appetite, whereas milk chocolate odor increased pleasantness.
- Lower hunger and greater fullness were associated with better performance among participants.
- Effects were observed after overnight fasting, indicating a non-ingestive strategy to support training.

## 1 Introduction

Resistance exercise is a fundamental component of athletic training, but performance during resistance exercise may be limited by factors such as fasting, caloric restriction, and heightened hunger. Recent research suggests that appetite perception, beyond energy availability alone, may influence exercise outcomes in both endurance and resistance exercise contexts (Bin Naharudin et al., 2019; Naharudin et al., 2022; Stratton et al., 2025).

Notably, placebo manipulations that alter perceived satiety without changing energy intake have been shown to either enhance or impair exercise performance (Hurst et al., 2020; Naharudin et al., 2020). These findings suggest that appetite may function not only as a nutritional signal but also as a psychobiological factor related to exercise behavior and performance.

Among external appetite modulators, olfaction represents a particularly potent and underexplored pathway. The olfactory system has close connections with limbic and hypothalamic regions, linking odor perception to affective responses and appetite regulation (Rolls, 2023; Stark, 2024). Food-related odors can elicit bidirectional effects: some scents stimulate salivation, gastric secretion, and eating motivation, whereas others suppress the desire to eat and evoke satiety-related responses (Yeomans, 2006; Fine and Riera, 2019; Lasschuijt et al., 2020)—for instance, sweet, palatable odors such as milk chocolate are associated with appetite stimulation, whereas bitter or less palatable odors, such as dark chocolate, may induce satiety and decrease intake (Sørensen and Astrup, 2011; Ramaekers et al., 2014; Morquecho-Campos et al., 2020). These effects may emerge rapidly, but they are also likely to depend on contextual factors such as prior experience, reward associations, and the duration or repetition of odor exposure (Brunstrom et al., 2011; Zoon et al., 2016; Biswas and Szocs, 2019; Stark, 2024).

While olfactory stimulation has been investigated in isolation for its effects on appetite (Yeomans, 2006; Massolt et al., 2010) or mood and motor performance (Raudenbush et al., 2001; Sowndhararajan et al., 2015; Barker et al., 2003), no study to date has systematically examined the triadic interaction among olfaction, appetite, and exercise performance. This gap is especially relevant in contexts such as intermittent fasting or pre-competition caloric restriction, where strategies to optimize performance without nutritional intake are of high practical value (Chambers et al., 2009; Bin Naharudin et al., 2019). Harnessing olfactory cues to modulate appetite could provide athletes with a low-cost, easily implementable method to enhance motivation and endurance, without the gastrointestinal burden of pre-exercise feeding (De Oliveira et al., 2014); evidence on intermittent fasting and performance further underscores the need for such non-ingestive approaches (Conde-Pipó et al., 2024).

Therefore, this study aimed to investigate whether controlled olfactory stimulation can modulate resistance exercise performance through appetite perception. Using a repeated-measures crossover design, the participants were exposed to distinct odorants (dark chocolate, milk chocolate, and control), while their appetite, pleasantness, and leg extension performance were assessed. We further explored whether appetite- and pleasantness-related responses were associated with performance outcomes, providing preliminary evidence on how food-related odors may shape resistance exercise responses in the fasted state. Elucidating this mechanism may position olfactory stimulation as a practical, non-ingestive sports nutrition adjunct to optimize exercise performance under dietary constraints. It may also deepen our understanding of the psychobiological determinants of resistance exercise.

## 2 Materials and methods

### 2.1 Participants

This study was conducted in accordance with the Declaration of Helsinki and was approved by the Universiti Malaya Research Ethics Committee (UM.TNC2/UMREC_3457). Written informed consent was obtained from all participants prior to enrollment. The study was preregistered on the Open Science Framework (OSF; DOI: https://doi.org/10.17605/OSF.IO/GU4E6) and retrospectively registered with the University Hospital Medical Information Network Clinical Trials Registry (UMIN-CTR; ID: UMIN000059639; registered on November 4, 2025; https://center6.umin.ac.jp/cgi-bin/ctr/ctr_view_reg.cgi?recptno=R000068210).

A total of 23 healthy men were recruited, all of whom completed the experimental trials (age 23.4 ± 2.1 years; body mass 72.7 ± 7.7 kg; height 176.2 ± 6.3 cm; BMI 22.4 ± 1.9 kg/m²). For enrollment into the study, the participants had to be nonsmokers, habitually consume breakfast, have no aversion to cocoa-related or sweet food odors, and regularly engage in resistance training (≥2 sessions per week for at least the previous 2 years). The exclusion criteria were self-reported olfactory dysfunction, substance abuse, metabolic or cardiovascular disease, and musculoskeletal injuries that could impair exercise performance.

The sample size for this study was estimated *a priori* using G*Power 3.1.9.6. Assuming a within-subject (paired-samples) comparison (two-tailed), *α* = 0.05, power (1 – *β*) = 0.80, and an expected effect size dz = 0.65, at least 22 participants were required to detect the anticipated effect; accordingly, 23 were recruited.

### 2.2 Study design

This was a randomized, double-blind, crossover trial. Odor conditions were counterbalanced across participants: 90% dark chocolate (90DC), milk chocolate (60MC), and control (CON; distilled water); all stimuli contained propylene glycol (PG) as the solvent. The participants completed five laboratory visits, including a 10-repetition maximum (10RM) assessment, a familiarization session, and three experimental trials scheduled at the same time of the day for each participant. The trials were separated by at least 4 days to minimize carryover effects. Each trial comprised two sequential phases under the same odor condition. The condition order was determined using a computer-generated Latin square randomization table. The allocation schedule was held and managed by a staff member who was not involved in participant testing. Before each visit, this staff member prepared the assigned stimulus in accordance with the randomization code, while the participants and the performance assessors remained blinded throughout the study.

The primary performance outcome was total repetitions across all sets. The secondary outcomes included the number of sets completed, repetitions per set, ratings of perceived exertion (RPE), and appetite-related measures (hunger, fullness, desire to eat, prospective food consumption [PFC]) plus odor pleasantness.

### 2.3 Procedures

#### 2.3.1 Preliminary and familiarization visits

During the first visit, the participants performed a 5-min cycling warm-up at 1.5 W/kg body mass followed by a 5-min self-selected warm-up before completing the 10-repetition maximum (10RM) test on the leg extension machine. The initial load was estimated from the participants’ training history and progressively adjusted until they were unable to complete 10 repetitions, with ≥3-min rest between attempts. The last successfully completed load was recorded as the 10RM and used to prescribe the experimental exercise load of 80%10RM. At the second visit, the participants were familiarized with all experimental procedures, including odor exposure, appetite ratings, and the exercise protocol.

#### 2.3.2 Experimental trials

**Figure 1:**
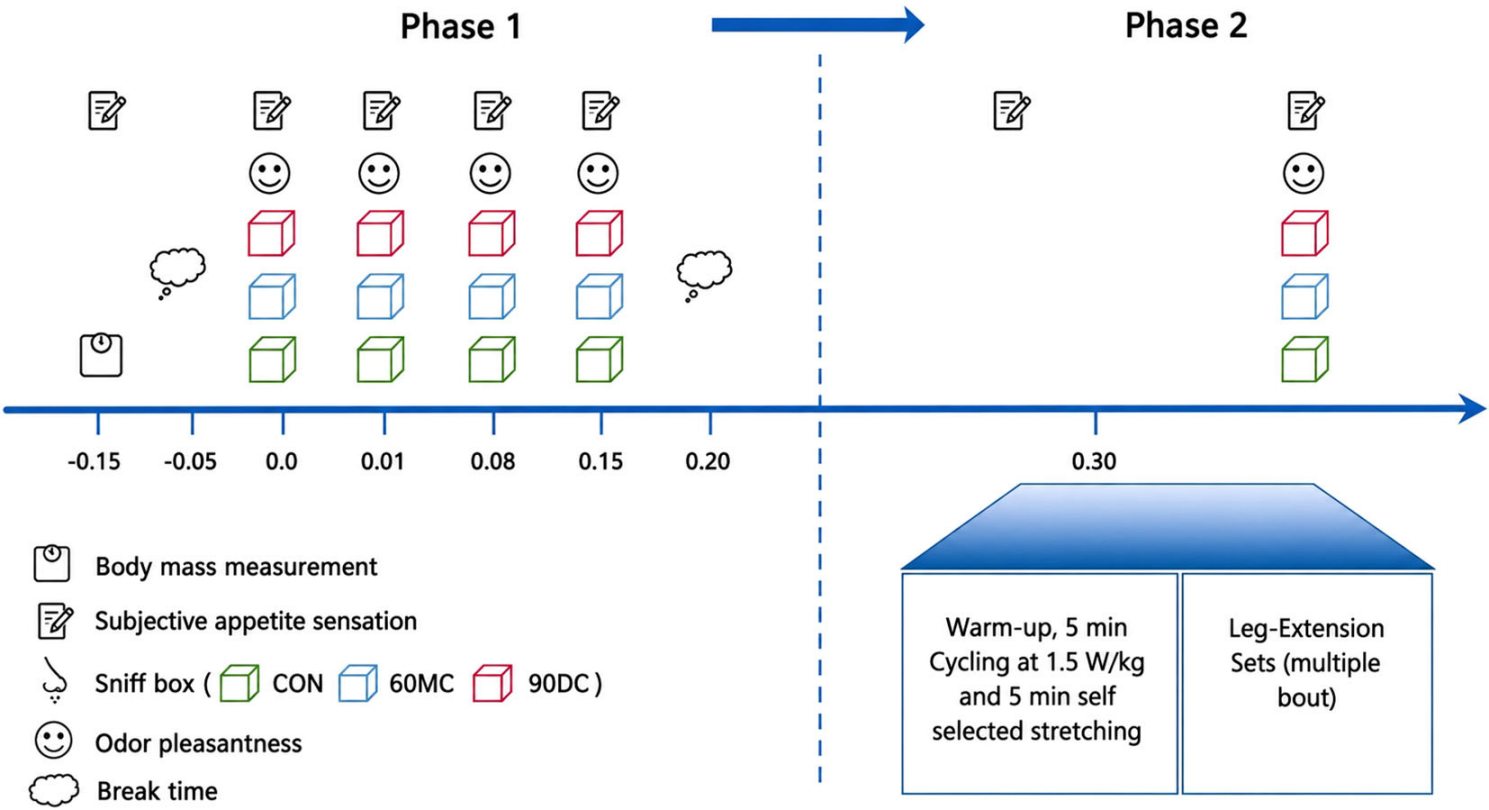
Experimental timeline and measurement schedule for the randomized, double-blind, crossover trial. Pre-exercise VAS were obtained at baseline (no exposure) and at *t* = 0, 1, 8, and 15 min, each immediately after a 30-s odor exposure. During exercise, the participants performed multiple-bout leg extension at 80% 10RM; each inter-set rest included a 30-s exposure followed by VAS (hunger, desire to eat, pleasantness). The conditions (90DC, 60MC, and CON) were counterbalanced.

The participants were instructed to avoid strenuous physical activity and alcohol for at least 72 h, as well as caffeine, nicotine, and scented products for at least 24 h before each trial. All sessions were conducted between 08:00 and 09:00 under standardized laboratory conditions following an overnight fast of at least 10 h. Upon arrival, body mass was measured and baseline appetite ratings were obtained using the 100-mm visual analog scales (VAS). Odor exposure was standardized across trials using identical sample jars, a fixed jar-to-nostril distance of approximately 2 to 3 cm, paced breathing (3-s inhalation and 3-s exhalation), and a fixed exposure duration of 30 s. At each visit, the odor condition was administered according to the pre-determined counterbalanced schedule (**Figure 1**).

#### 2.3.3 Blinding and blinding assessment (Bang’s BI)

Odor solutions were prepared and code-labeled (A/B/C) by an independent staff member using identical containers and standardized handling. The participants and repetition-count assessors were blinded to odor identity; the assessors did not smell the stimuli and had no access to the code–condition mapping. The data analysts remained blinded until completion of the primary analysis.

After all of the three visits, the participants retrospectively indicated, for each visit, which coded condition (A/B/C or “don’t know”) they believed had been delivered and provided a brief rationale; forms were submitted before debriefing. We constructed an actual × guessed contingency table using the participants’ responses only. We computed Bang’s blinding index (BI) separately for each condition (90DC, 60MC, and CON) with two-sided 95% bootstrap confidence intervals (Bang et al., 2004). “Don’t know” responses were summarized descriptively and excluded from BI computation. Masking was considered adequate *a priori* when the 95% CI included 0; *p*-values tested deviation from chance guessing of 1/3. Given the odorless water control, the CON BI was reported descriptively.

#### 2.3.4 Olfactory stimulant preparation

Three liquid odor stimuli were prepared to a final volume of approximately 40 mL per bottle and presented in identical glass jars (diameter, 5 cm). Propylene glycol (PG; 5% v/v) was included in all stimuli to aid dispersion and to standardize volatility across conditions. The ingredients were weighed by mass.

For 90DC, commercial 90% cocoa dark chocolate was melted in a warm water bath (~40 °C) and mixed with distilled water and PG on a magnetic stirrer until uniform (suspension). For 60MC, commercial dark chocolate and whole milk were combined with distilled water and PG and mixed on a magnetic stirrer for 3–5 min to obtain a uniform emulsion. The control stimulus consisted of distilled water and PG prepared with the same procedures. The preparations were aliquoted to ~40 mL per bottle. Immediately before each exposure, the bottles were gently inverted three to five times to re-suspend the mixture. A visual inspection confirmed the absence of visible phase separation over the testing window. Detailed formulations and proportions are summarized in **Table 1**.

**Table 1:** Composition of olfactory stimuli (per ~40-mL solution).

Condition	Ingredients	Final volume (mL)	Notes
90DC (dark chocolate)	Dark chocolate 36 g (melt volume 34.2 mL at ~40 °C; Benns Ethicoa, Kuala Lumpur, Malaysia) + distilled water 3.8 mL + PG 2.0 mL	~40	Suspension
60MC (milk chocolate)	Dark chocolate 12 g (melt volume 11.4 mL at ~40°C; Benns Ethicoa, Kuala Lumpur, Malaysia) + whole milk 22.8 mL (Dutch Lady, Petaling Jaya, Malaysia; 3.2% fat) + distilled water 3.8 mL + PG 2.0 mL	~40	Emulsion
Con (control)	Distilled water 38.0 mL + PG 2.0 mL	~40	Solvent only

Melt volume denotes the displaced volume of melted chocolate measured at ~40 °C and was used solely to reach the target volume (~40 mL); chocolate was weighed by mass, and no mass-to-volume conversion was assumed.

PG, propylene glycol (5% v/v, volatility standardizer).

### 2.4 Outcome measures

#### 2.4.1 Subjective appetite perception

Appetite was measured with 100-mm visual analog scales (VAS) for hunger, fullness, desire to eat, and prospective food consumption (PFC). Scales were anchored at 0 mm (“not at all”) to 100 mm (“extremely”). Pre-exercise ratings were recorded at baseline and *t* = 0, 1, 8, and 15 min, each immediately after a 30-s exposure. During exercise, only hunger and desire to eat were recorded during each set after a 30-s exposure.

#### 2.4.2 Emotional ratings

Odor pleasantness was rated on a 100-mm VAS using the same anchors. The ratings were recorded at baseline and at 0, 1, 8, and 15 min in the pre-exercise phase (each immediately after a 30-s exposure) and during inter-set rest in the exercise phase.

#### 2.4.3 Performance outcomes

Resistance exercise performance was assessed during a multi-set leg extension task performed at 80% of the individually determined 10RM. The primary outcome was the total number of valid repetitions completed across all sets. The secondary outcomes were the number of sets completed and repetitions per set. RPE (Borg CR10) was recorded after each set. Technical standardization (seat and pad alignment, metronome-paced cadence, and repetition validity) followed the procedures.

### 2.5 Statistical analyses

All outcomes were analyzed with linear mixed-effects models (LMMs). Each model included fixed effects for condition (90DC, 60MC, CON), a within-trial time factor, the condition × time/set interaction, and a participant random intercept. In phase 1, the time factor was measurement time (*t* = 0, 1, 8, and 15 min; baseline used only as a covariate) and was treated as categorical. In phase 2, set number was modeled as a continuous linear term. For VAS outcomes, the condition-specific baseline value of the same scale was included as a covariate to account for pre-exposure differences between trials. For repeated VAS and set-level outcomes, residuals were modeled using a first-order autoregressive [AR (1)] covariance structure within each participant × condition combination, reflecting the ordered nature of the repeated measurements. For outcomes measured once per condition, such as total repetitions and total sets, within-participant dependence was modeled using a participant random intercept.

Models were fitted by restricted maximum likelihood. Fixed effects were tested with type III Wald *χ*² tests derived from the LMMs, and the reported *χ*² statistics reflect tests of model terms. If the condition × time/set interaction was significant, Bonferroni-adjusted marginal means were compared within the relevant time point or set; otherwise, Bonferroni-adjusted main-effect marginal means were compared. Model-based mean differences (Δ) are reported with 95% confidence intervals, and standardized contrasts are reported as Cohen’s *d*. Tests were two-tailed with *α* = 0.05.

Exploratory mechanism-related analyses were also conducted. First, piecewise mixed-model mediation analyses were used to examine three theory-driven pathways: 90DC versus CON through pre-exercise hunger, 90DC versus CON through pre-exercise fullness, and 60MC versus CON through pre-exercise odor pleasantness. Total repetitions were used as the performance outcome. Indirect effects were calculated as the product of the a and b paths, with approximately 95% confidence intervals estimated using the product-of-coefficients approach. These mediation analyses were considered exploratory and hypothesis-generating. Second, repeated-measures correlation analyses were conducted to describe within-subject associations between pre-exercise subjective ratings and performance outcomes across conditions. These analyses focused on hunger, fullness, and odor pleasantness in relation to total repetitions and total sets and were interpreted as exploratory associations rather than evidence of mediation or causality.

Analyses were performed using Python (version 3.13.7; statsmodels), SPSS, and R. Repeated-measures correlations were performed using the rmcorr package in R.

## 3 Results

### 3.1 Participants and baseline characteristics

All 23 participants completed whole trials. The pre-trial dietary controls did not differ between conditions; further details are shown in **Table 2**.

**Table 2:** Pre-trial dietary controls by condition.

Variable	90DC	60MC	CON
Energy intake (kcal)	2,514.5 ± 169.8	2,481.6 ± 191.3	2,427.5 ± 177.8
Fasting time (h)	9.9 ± 0.6	10.1 ± 0.8	10.0 ± 0.6

Values are mean ± SD (*n* = 23). Energy intake and fasting time did not differ between conditions (LMM: energy intake, *p* > 0.05; fasting time, *p* = 0.565).

90DC, 90% dark chocolate; 60MC, milk chocolate; CON, control; kcal, kilocalories; h, hours.

### 3.2 Subjective appetite sensations (phase 1)

**Figure 2:**
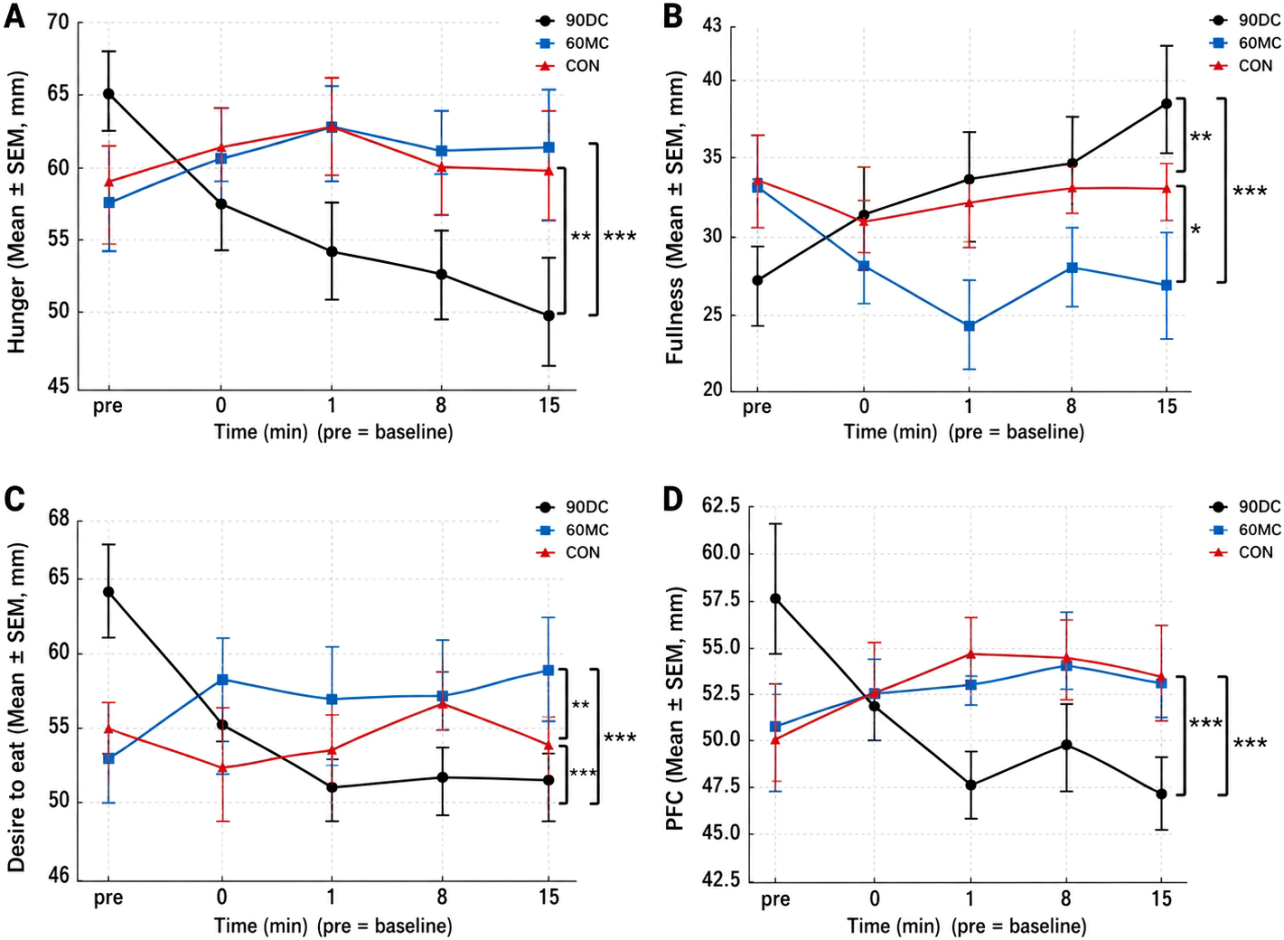
Subjective appetite during the pre-exercise phase: **(A)** hunger, **(B)** desire to eat, **(C)** fullness, and **(D)** prospective food consumption. Points show baseline-adjusted estimated marginal means (EMMs) ± model-based SE at 0, 1, 8, and 15 min. Baseline values are displayed descriptively only and were included as covariates in the LMM. Symbols: ● 90DC, ■ 60MC, ▲ CON. Brackets indicate Bonferroni-adjusted pairwise contrasts of model-based marginal means averaged over 0 -15 min; *p < 0.05, **p < 0.01, ***p < 0.001. Error bars represent model-based SE for visualization; inferential statistics are based on baseline-adjusted LMMs.

Across 0–15 min, there was a clear condition effect on all four scales (hunger, desire to eat, fullness, PFC; all *p* < 0.001), with no effects of time or condition × time (*p* ≥ 0.30). Relative to CON, 90DC consistently produced lower hunger and desire to eat and higher fullness, and it also reduced PFC (all *p* ≤ 0.001). The same pattern held for 90DC vs. 60MC (all *p* < 0.001). In contrast, 60MC differed from CON only for desire to eat (higher with 60MC; *p* = 0.008), with no differences on the other scales. Descriptive means ± SEM are shown in **Figures 2A–D**.

### 3.3 Odor pleasantness (phase 1)

**Figure 3:**
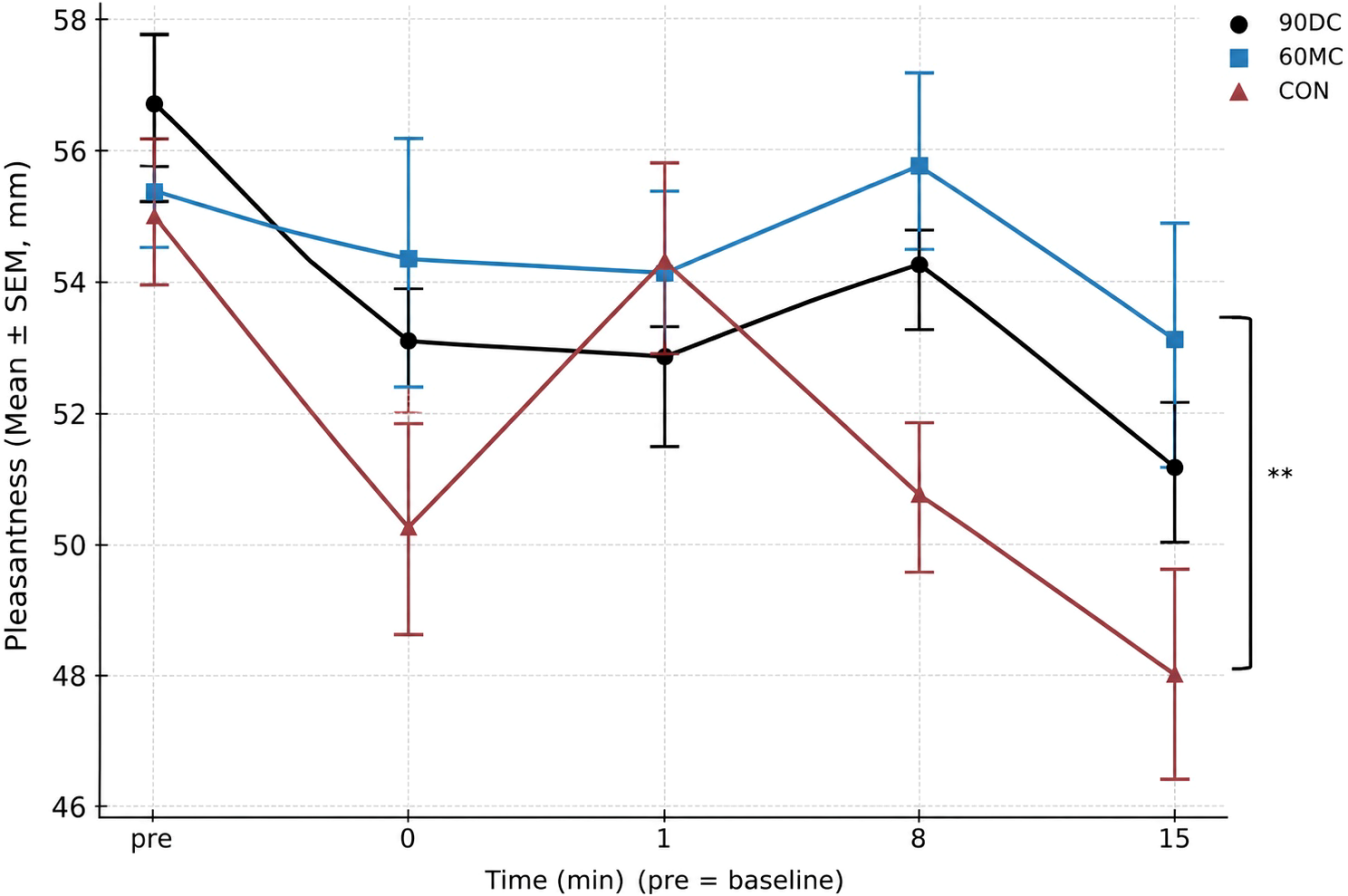
The revised caption should read as follows: Odor pleasantness during the pre-exercise phase. Points show baseline-adjusted estimated marginal means (EMMs) ± model-based SE on a 0 -100 mm VAS at 0, 1, 8, and 15 min. Baseline values are displayed descriptively only and were included as covariates in the LMM. Symbols: ● 90DC, ■ 60MC, ▲ CON. Brackets indicate Bonferroni-adjusted pairwise contrasts of model-based marginal means averaged over 0 -15 min; **p < 0.01. Error bars represent model-based SE for visualization; inferential statistics are based on baseline-adjusted LMM.

A baseline-adjusted linear mixed-effects model showed a main effect of condition (Wald *χ*²(2) = 8.99, *p* = 0.011). Averaged across 0–15 min (model-based marginal means, baseline-adjusted), 60MC was rated more pleasant than CON (Δ = 3.10 mm, 95% CI 1.06–5.14; *p* = 0.009), whereas 90DC did not differ from CON or 60MC (*p* = 0.660 and *p* = 0.246). The main effects of time and the condition × time interaction were not significant (time: *p* = 0.051; condition × time: *p* = 0.490). Descriptive means ± SEM are depicted in **Figure 3**.

### 3.4 Performance outcomes

The baseline-adjusted linear mixed-effects models showed a condition effect for total repetitions (Wald *χ*²(2) = 71.15, *p* < 0.001) and total sets (Wald *χ*²(2) = 21.27, *p* < 0.001).

Total repetitions: The participants completed 18 more repetitions with 90DC than CON (95% CI 13.8–22.1; *p* < 0.001) and nine more with 60MC than CON (95% CI 4.8–13.1; *p* < 0.001); 90DC also exceeded 60MC by nine repetitions (95% CI 4.8–13.2; *p* < 0.001).

Total sets: 90DC resulted in one more set than CON (95% CI 0.7–1.8; *p* < 0.001) and one more set than 60MC (95% CI 0.4–1.5; *p* = 0.003), while 60MC and CON were similar (*p* = 0.804; 95% CI for the difference -0.2–0.8).

RPE increased progressively across sets (*p* < 0.001). Neither the main effect of condition nor the condition × set interaction reached significance (*p* ≥ 0.31). Thus, perceived exertion rose with task progression in all conditions, with no evidence of odor-related differences.

**Figure 4:**
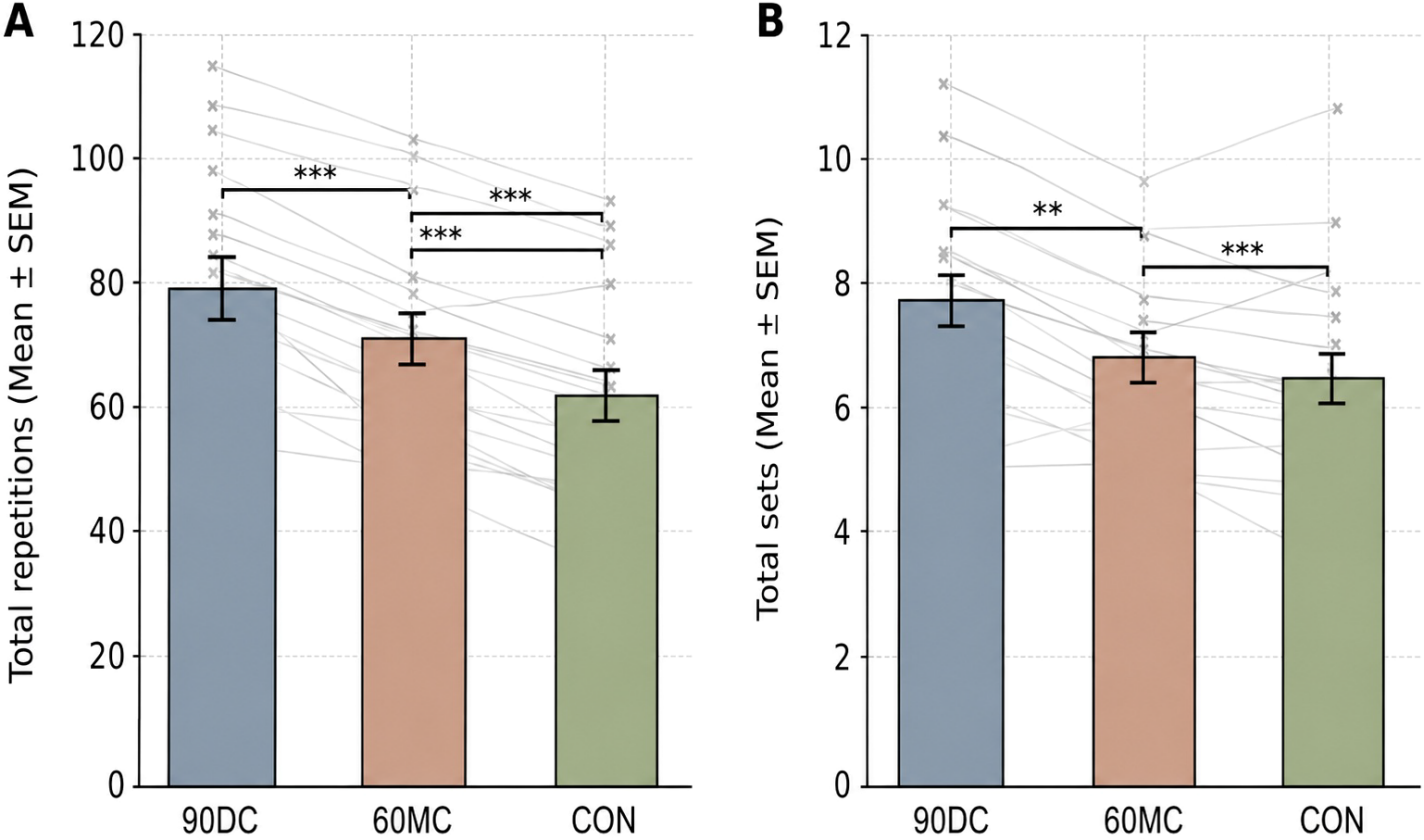
Performance under three conditions. **(A) **Total repetitions and **(B)** total sets are shown as model-estimated marginal means ± model-based SE. Thin gray lines indicate paired participant data across conditions. Brackets denote Bonferroni-adjusted pairwise contrasts of model-based marginal means; **p < 0.01, ***p < 0.001. Error bars represent model-based SE for visualization; inferential statistics are based on linear mixed-effects models with a participant random intercept.

Descriptive means ± SEM are shown in **Figures 4A, B**.

### 3.5 Subjective appetite sensations (phase 2)

Hunger was lower with 90DC than with 60MC and CON across sets (both *p* < 0.001), and 60MC was lower than CON (*p* = 0.016). Hunger also declined over sets, with a small condition × set interaction (*p* = 0.020).

**Figure 5:**
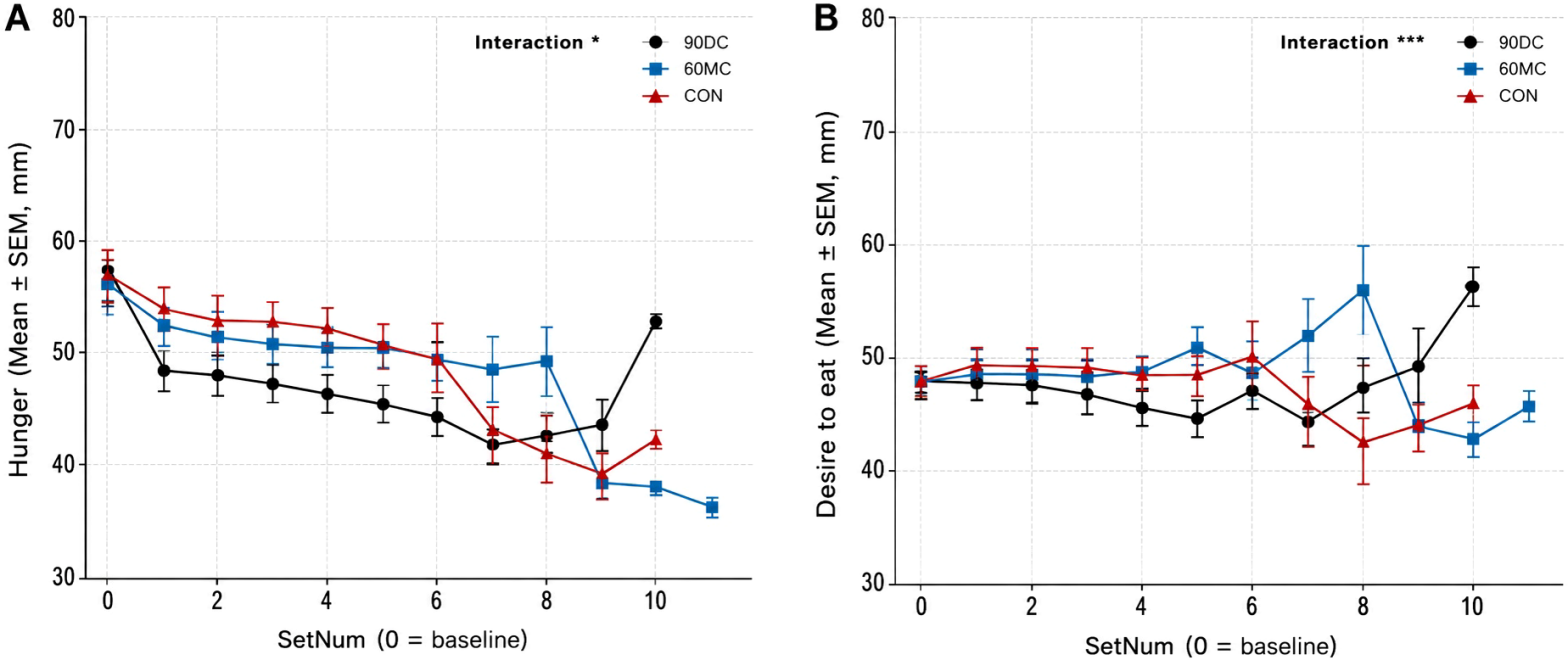
Hunger **(A)** and desire to eat **(B)** during the leg extension task. The points show model-estimated marginal means ± model-based SE at each inter-set rating. Pre-exercise baseline values are displayed descriptively only and were included as covariates in the LMM. ●, 90DC; ■, 60MC; ▲, CON. Short annotations above the panels indicate the condition × set interaction: *p* < 0.05, *p* < 0.01, *p* < 0.001. The interaction reflected greater separation among condition-specific trajectories toward the later part of the exercise task. Error bars represent model-based SE for visualization; inferential statistics reflect Bonferroni-adjusted contrasts of model-based marginal means from the LMMs.

Desire to eat was lower with 90DC than with 60MC and CON across sets (both *p* < 0.001), whereas 60MC and CON were similar (*p* = 1.000). There was no overall effect of set, but the condition × set interaction was significant (*p* < 0.001). Because the participants completed different numbers of sets before reaching volitional exhaustion, pairwise contrasts were interpreted as Bonferroni-adjusted condition differences averaged across sets rather than set-specific comparisons. Descriptive means ± SEM are shown in **Figures 5A, B**.

### 3.6 Odor pleasantness (phase 2)

**Figure 6:**
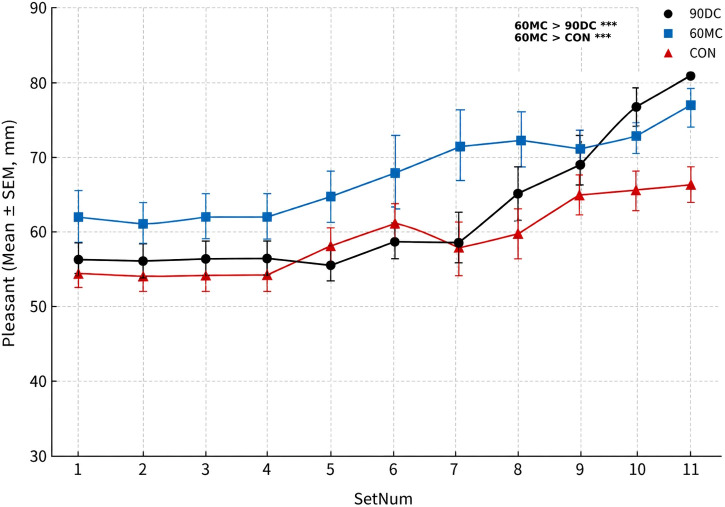
Odor pleasantness during the leg extension task. The points show model-estimated marginal means ± model-based SE on a 0–100-mm VAS at each inter-set rating. ●, 90DC; ■, 60MC; ▲, CON. Plot annotations denote Bonferroni-adjusted pairwise contrasts of model-based marginal means averaged across sets because the condition × set interaction was not significant. *p* < 0.05, *p* < 0.01, *p* < 0.001. Error bars represent model-based SE for visualization; inferential statistics are based on linear mixed-effects models.

There was a condition effect (*p* < 0.001), while SET AND CONDITION × SET were not significant (*p* = 0.781 and *p* = 0.055). Across sets, 60MC was rated more pleasant than both 90DC and CON (both *p* < 0.001); 90DC and CON did not differ. Descriptive means ± SEM are shown in **Figure 6**.

### 3.7 Exploratory mediation and repeated-measures correlation analyses

First, exploratory mediation analyses were performed using a piecewise mixed-model approach. For the 90DC versus CON contrast, 90DC was associated with lower pre-exercise hunger (*a* = −13.17, SE = 3.29, *p* < 0.001). The hunger–performance path was negative but not statistically clear (*b* = −0.17, SE = 0.13, *p* = 0.199), and the indirect effect through hunger was positive but inconclusive (*ab* = 2.24 repetitions, 95% CI −1.25 to 5.73, *p* = 0.208). For the fullness model, 90DC showed a non-significant tendency toward higher fullness (*a* = 6.57, SE = 4.13, *p* = 0.119), the fullness–performance path was positive but not statistically clear (*b* = 0.12, SE = 0.09, *p* = 0.212), and the indirect effect was inconclusive (*ab* = 0.78 repetitions, 95% CI −0.75 to 2.31, *p* = 0.317). For the 60MC versus CON contrast, 60MC was associated with higher pleasantness (*a* = 3.10, SE = 0.91, *p* = 0.003), but neither the pleasantness–performance path (*b* = 0.31, SE = 0.37, *p* = 0.420) nor the indirect effect (*ab* = 0.95 repetitions, 95% CI −1.38 to 3.29, *p* = 0.424) was statistically clear. These exploratory analyses did not provide clear statistical evidence that hunger, fullness, or pleasantness mediated condition-related differences in total repetitions (**Table 3**). Nevertheless, the hunger and fullness models showed directionally consistent patterns with the proposed appetite-related interpretation, whereas the pleasantness-related pathway was less clearly supported.

**Table 3:** Exploratory multilevel mediation analyses.

Model	Contrast	Mediator	*a* path	*b* path	Indirect effect	95% CI	*p*
1	90DC vs. CON	Hunger	-13.17	-0.17	2.24	-1.25 to 5.73	0.208
2	90DC vs. CON	Fullness	6.57	0.12	0.78	-0.75 to 2.31	0.317
3	60MC vs. CON	Pleasantness	3.10	0.31	0.95	-1.38 to 3.29	0.424

Indirect effects were calculated as *ab*. Confidence intervals were estimated using the product-of-coefficients approach. These analyses were exploratory and hypothesis-generating.

*a* path, effect of condition on the proposed mediator; *b* path, within-participant association between the subject-centered mediator and total repetitions while retaining condition in the model.

**Figure 7:**
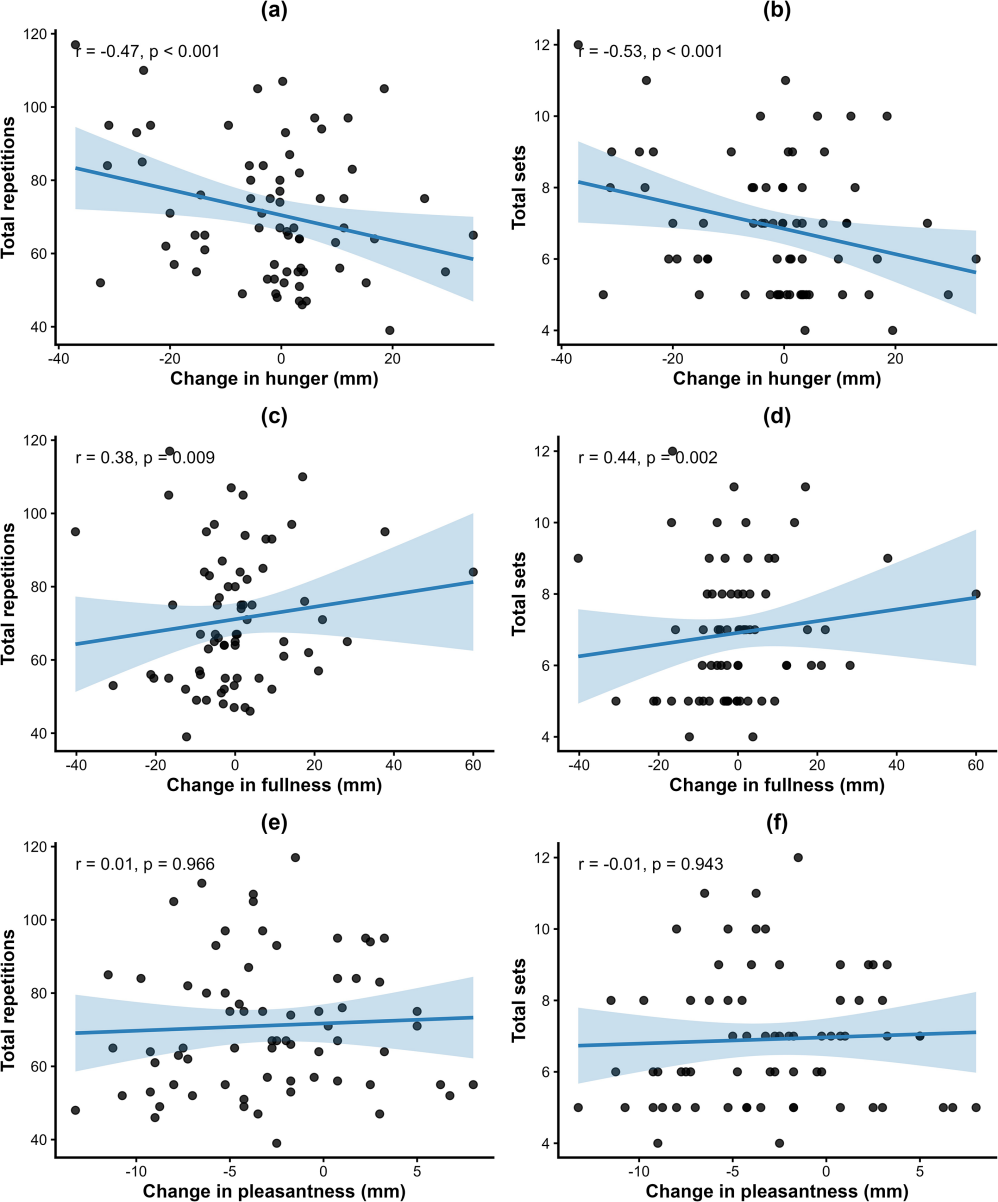
Repeated-measures correlations between subjective ratings and resistance exercise performance. The panels show within-participant associations between change in hunger and **(a)** total repetitions and **(b)** total sets, change in fullness and **(c)** total repetitions and **(d)** total sets, and change in odor pleasantness and **(e)** total repetitions and **(f)** total sets. The points represent participant-by-condition observations. The lines indicate repeated-measures correlation fits with 95% confidence bands. The panel annotations report repeated-measures correlation coefficients (rrm) and two-tailed *p*-values.

Repeated-measures correlation analyses were then conducted to describe within-subject associations between pre-exercise subjective responses and performance outcomes. As shown in **Figure 7**, hunger was inversely associated with both total repetitions (rrm = −0.47, 95% CI −0.67 to −0.21, *p* < 0.001) and total sets (rrm = −0.53, 95% CI −0.71 to −0.29, *p* < 0.001). In contrast, fullness was positively associated with total repetitions (rrm = 0.38, 95% CI 0.10 to 0.60, *p* = 0.009) and total sets (rrm = 0.44, 95% CI 0.18 to 0.65, *p* = 0.002). Pleasantness was not associated with either total repetitions (rrm = 0.01, *p* = 0.966) or total sets (rrm = −0.01, *p* = 0.943). Thus, although statistical mediation was not confirmed, lower pre-exercise hunger and greater pre-exercise fullness were associated with higher performance outcomes within participants, whereas pleasantness was not associated with performance.

### 3.8 Blinding assessment

Across 69 observations (23 participants × 3 conditions), Bang’s blinding index (BI; 95% CI) by condition was 90DC 0.22 (-0.10–0.54), 60MC 0.28 (-0.04–0.60), and CON 0.87 (0.58–0.98). As prespecified, masking for odor conditions was considered adequate when the 95% CI included 0; this criterion was met for both 90DC and 60MC. Given the odorless control, CON showed greater identifiability and is reported descriptively. Detailed indices are reported in **Table 4**.

**Table 4:** Bang’s blinding index (BI) by condition.

Condition	BI	95% CI	*p* value (vs 1/3)	*n* (trials)	% “Don’t know”
90DC	0.22	-0.10 - 0.54	0.1827	23	21.7
60MC	0.28	-0.04 - 0.60	0.0745	23	21.7
CON	0.87	0.58 - 0.98	0.0000	23	0

BI ranges from −1 to +1; 0 ≈ chance. “Don’t know” is reported but excluded from BI.

*n* (trials), participants per condition; positive, greater identifiability; negative, opposite-than-chance.

## 4 Discussion

The present study provides preliminary evidence that chocolate-related olfactory stimulation can increase resistance exercise volume during a fasted, single-joint leg extension task while altering appetite-related and pleasantness responses. Specifically, under the 90DC condition, the participants reported lower hunger, desire to eat, and prospective food consumption, together with greater fullness before exercise. Similar condition-specific differences were also observed during inter-set rest, although these ratings were obtained shortly after exercise bouts and may therefore reflect both olfactory and exercise-induced influences. These appetite-related responses were accompanied by greater training volume, without clear increases in post-set ratings of perceived exertion (RPE). In contrast, under the 60MC condition, the participants consistently reported higher odor pleasantness before and during exercise, whereas appetite suppression was not observed. Despite these distinct subjective profiles, 60MC also increased total repetitions without clear changes in post-set RPE. Taken together, these findings suggest that food-related odors may be associated with greater resistance exercise volume under fasted conditions, alongside changes in subjective appetite and odor pleasantness before and during exercise. The response to 90DC appeared to be more closely aligned with reduced appetite-related sensations, whereas the response to 60MC appeared to be characterized primarily by a more pleasant olfactory experience.

This interpretation is consistent with previous work linking appetite perception to resistance exercise performance, particularly under fasting conditions. In a randomized crossover trial, Bin Naharudin et al. (2019) reported that omitting breakfast reduced subsequent resistance exercise repetitions and was accompanied by greater hunger and lower satiety, whereas pre-exercise feeding attenuated hunger and improved performance. In a follow-up study, Naharudin et al. (2020) showed that both a high-viscosity placebo breakfast and an iso-flavor, iso-texture high-carbohydrate breakfast similarly reduced hunger, increased satiety, and improved squat repetitions compared with water, suggesting that early satiety-related responses may contribute to performance even when energy provision differs (Bin Naharudin et al., 2019; Naharudin et al., 2020). This interpretation is also consistent with broader evidence showing that texture and viscosity can enhance satiety (Stribiţcaia et al., 2020) and with findings from carbohydrate mouth-rinse studies indicating that oral sensory cues can alter exercise performance in the absence of nutrient ingestion (Chambers et al., 2009). Moreover, Naharudin et al. (2022) further demonstrated that lower pre-exercise hunger was associated with better back squat performance, reinforcing the appetite–performance link in resistance exercise contexts (Naharudin et al., 2022).

Against this background, the findings observed under the 90DC condition are particularly noteworthy. Despite the absence of nutrient ingestion, exposure to the 90DC odor alone reduced hunger and the desire to eat, increased fullness, and improved training volume. These findings suggest that 90DC odor may have transiently altered subjective appetite perception under fasted conditions, shifting the participants from a state of heightened hunger toward a relatively greater sense of satiety. This shift may have reduced the potential interference of internal hunger-related sensations with engagement in the exercise task. This interpretation is consistent with the direction of the exploratory mediation estimates and is further supported by repeated-measures correlation analysis, which showed that lower hunger and greater fullness were associated with better exercise performance.

This effect may partly reflect expectancy effects or cue-driven anticipatory responses. Previous studies have shown that mindsets and expectations can influence ghrelin responses independently of actual nutrient content (Crum et al., 2011) and that placebo manipulations can alter subjective appetite and satiety perceptions (Hoffmann et al., 2018). Consistent with this, Massolt et al. (2010) reported that both consuming dark chocolate and smelling dark chocolate odor increased satiety and reduced hunger (Massolt et al., 2010). The 90DC response may therefore be interpreted within a broader framework of learned odor–food associations, expectancy, and cephalic-phase physiology. Food odors are biologically salient cues because olfactory information is closely connected with neural systems involved in reward evaluation, memory, emotion, and appetite regulation, including limbic, orbitofrontal, and hypothalamic networks. In the present study, 90DC odor may have acted as a learned cue associated with a rich, bitter, and relatively satiating chocolate product. Through previous eating experiences, such cues may generate expectations about the sensory and post-ingestive consequences of food, thereby shifting subjective appetite from hunger toward fullness.

Such cue-driven responses may also involve cephalic-phase processes. Cephalic-phase responses are anticipatory physiological changes elicited by sensory food cues before nutrient absorption and are thought to prepare the organism for potential ingestion and digestion. These responses may include salivation, gastric activity, autonomic adjustments, and endocrine changes involving insulin, ghrelin, pancreatic polypeptide, GLP-1, PYY, or CCK (Power and Schulkin, 2008; Lasschuijt et al., 2020; Pullicin et al., 2021). Therefore, the lower hunger and greater fullness observed under 90DC before exercise may plausibly reflect an odor-related anticipatory appetite response rather than a response to nutrient ingestion.

Nevertheless, this interpretation remains preliminary because expectancy, salivation, gastric activity, autonomic function, appetite-related hormones, and neural responses were not directly measured. Accordingly, the proposed cephalic-phase and expectancy-related mechanisms should be viewed as plausible explanatory frameworks rather than confirmed pathways. This distinction is particularly important for appetite ratings obtained during the exercise phase because these responses may have been influenced by the exercise task itself in addition to odor exposure.

Acute exercise itself can transiently suppress appetite, a phenomenon commonly described as exercise-induced appetite suppression or exercise-induced anorexia. This response has been associated with short-term changes in appetite-regulating hormones, including reduced acylated ghrelin and increased anorexigenic peptides such as peptide YY and glucagon-like peptide-1, although the magnitude and timing of these responses vary according to exercise intensity, modality, duration, nutritional status, and participant characteristics (Stensel, 2010; Douglas et al., 2017; Thackray and Stensel, 2023; McCarthy et al., 2024). In resistance exercise, appetite-related responses may also be influenced by training load, total volume, and inter-set recovery, and the evidence remains less consistent than that observed after aerobic exercise (Broom et al., 2009; Halliday et al., 2021; Liu et al., 2023). In the present study, the pre-exercise phase provides the clearest evidence for an odor-related appetite response because appetite ratings were obtained before the leg extension task began. By contrast, inter-set appetite ratings were obtained shortly after repeated exercise bouts and therefore may reflect the combined effects of odor exposure, exercise-induced appetite modulation, fatigue, respiratory recovery, and attentional demands. Thus, the during-exercise appetite findings should be interpreted as condition-specific subjective responses during exercise rather than as evidence that odor exposure alone suppressed appetite during the task.

A distinct subjective response profile emerged under the 60MC condition. In this condition, greater exercise volume was observed without consistent suppression of appetite but alongside higher pleasantness ratings both before and during exercise. This pattern is broadly consistent with the Specific Sensory Appetite framework, which proposes that food-related sensory cues may selectively enhance the desire for sensory-congruent qualities without necessarily changing general hunger (Ramaekers et al., 2014; Zoon et al., 2016; Morquecho-Campos et al., 2020). Previous studies have similarly shown that sweet or chocolate-like odors can increase wanting and shift preferences toward congruent foods (Sørensen and Astrup, 2011; Proserpio et al., 2017; Morquecho-Campos et al., 2020).

However, in the present study, pleasantness was not associated with total repetitions or total sets in the repeated-measures correlation analyses, and the indirect effect through pleasantness was not statistically clear. Therefore, pleasantness should be interpreted as a characteristic subjective response to 60MC rather than as evidence that hedonic-affective mechanisms explained the observed performance effect. Future studies incorporating measures of affect, motivation, autonomic regulation, and central fatigue are needed to determine whether pleasant odor contexts can influence exercise behavior through these processes.

The present findings also have practical implications for the timing and delivery of olfactory interventions. In this study, repeated brief odor exposures of 30 s were sufficient to alter subjective responses and improve exercise performance, suggesting that relatively short, strategically timed presentations may be adequate to induce meaningful psychobiological effects. Such brief exposures may operate through rapid cue-driven sensory and anticipatory processes (Zoon et al., 2016; Lasschuijt et al., 2020; Pullicin et al., 2021) while also limiting olfactory adaptation or habituation that can occur during continuous exposure (Ferdenzi et al., 2014; Pellegrino et al., 2017; Mignot et al., 2022). From an applied perspective, this is encouraging because short odor presentations before exercise and between sets are likely to be more feasible in real-world training settings than continuous odor administration. Evidence from olfactory priming research also suggests that even brief and incidental exposures can bias appetite-related responses and subsequent behavior in a cue-congruent manner (Ferriday and Brunstrom, 2011; Gaillet-Torrent et al., 2014).

These effects may be particularly relevant under fasted or energy-restricted conditions, where hunger-related signals are likely to be more salient. In the present study, the participants completed the protocol after 10–11 h of fasting, which may have amplified the influence of appetite-related and hedonic cues on subsequent performance. Under such conditions, appetite-suppressing odors such as 90DC may be especially useful when hunger represents a competing internal signal, whereas more pleasant and reward-linked odors such as 60MC may be more beneficial when affective or motivational support is the dominant pathway. However, whether the relative effectiveness of these odor types varies according to nutritional state, glycogen availability, or exercise modality remains to be tested directly.

Overall, these findings suggest that chocolate-related odors may be associated with greater resistance exercise volume under fasted conditions, alongside changes in subjective appetite and odor pleasantness. The response to 90DC appeared to be more closely aligned with reduced appetite-related sensations, whereas the response to 60MC appeared to be characterized primarily by a more pleasant olfactory experience. However, because the present analyses were exploratory and did not confirm statistical mediation, these findings should be interpreted as preliminary evidence rather than proof of causal psychobiological mechanisms.

## 5 Limitations and future directions

Several limitations should be acknowledged. First, the proposed psychobiological mechanisms remain inferential because no hormonal, autonomic, or neurophysiological measurements were included. Accordingly, the present study cannot determine whether appetite suppression, pleasantness, or other variables statistically mediated the observed performance effects. Although the pre-exercise appetite ratings provide the clearest evidence for an odor-related subjective response, appetite ratings collected during inter-set rest should be interpreted more cautiously. These ratings were obtained shortly after repeated exercise bouts and may therefore have been influenced not only by odor exposure but also by exercise-induced appetite suppression, fatigue, respiratory recovery, and attentional demands. Future studies should include serial measurements of appetite-related hormones, such as acylated ghrelin, GLP-1, and PYY, as well as exercise-only or expectancy-matched control conditions, to better distinguish odor-specific effects from exercise-induced appetite regulation.

Second, appetite, pleasantness, and perceived exertion were assessed using subjective rating scales. Although 100-mm visual analog scales and the Borg CR10 scale are commonly used in appetite and exercise research, these tools depend on the participants’ momentary perception, attention, and willingness to report accurately. This issue is particularly relevant for ratings obtained immediately after exercise sets, when fatigue, discomfort, altered breathing, and reduced attentional clarity may have influenced responses. Therefore, the inter-set subjective ratings should be interpreted cautiously and should be complemented in future studies by objective physiological, behavioral, and neuroendocrine measures.

Third, expectancy effects related to the control condition should be considered. Although Bang’s blinding index suggested acceptable masking for the two active odor conditions, the odorless CON condition was highly identifiable. Participants who recognized the absence of an active odor may have inferred that they were receiving the control or placebo condition, which could have reduced the positive expectancy or introduced a minor nocebo-like effect on subjective responses or exercise behavior. This issue is particularly relevant because placebo and nocebo effects have been shown to influence sport and exercise performance, with recent reviews indicating that expectations can produce small to moderate effects on performance outcomes (Hurst et al., 2020; Chhabra and Szabo, 2024). Therefore, differences between the active odor conditions and CON may partly reflect expectancy-related influences in addition to odor-specific sensory effects. Future studies should consider using an odorized placebo, a low-intensity neutral odor, or expectancy-matched control stimuli to better separate olfactory-specific effects from expectancy effects.

Fourth, odor delivery was performed using a standardized jar-based method rather than an olfactometer. Although jar-to-nostril distance, exposure duration, and breathing rhythm were controlled, this method did not allow the precise regulation of odor concentration, airflow, or the amount of volatile compounds reaching the nasal cavity. Minor variations in sniff depth, head position, stimulus mixing, or evaporation may therefore have introduced variability within and between participants. This limits reproducibility and prevents clear dose–response interpretation. Future studies should use olfactometer-based delivery or objective characterization of headspace volatile concentrations to improve stimulus consistency.

Fifth, the interpretation of the RPE findings should be considered cautiously. Although post-set RPE did not differ clearly between conditions, RPE was assessed only after each set, and no additional perceptual, motivational, neuromuscular, or central fatigue measures were included. Therefore, the absence of condition differences in post-set RPE should not be interpreted as evidence that odor exposure had no effect on broader perceptual or fatigue-related processes. Future studies should include complementary measures such as session RPE, affective valence, arousal, motivation ratings, neuromuscular fatigue indices, or neurophysiological measures to better characterize how olfactory cues may influence perceived effort and fatigue regulation.

Finally, generalizability is limited by the use of a single-joint leg extension task and a homogeneous sample of young resistance-trained men. The findings primarily apply to resistance exercise volume in this specific lower-limb task. Future studies should test whether similar responses occur across other exercise modalities, upper- and lower-body tasks, different nutritional states, and more diverse participant groups. They should also combine subjective measures with objective indices, such as appetite-related hormones, heart rate variability, electroencephalography, olfactory-event-related potentials, and functional neuroimaging, to directly test the mechanisms underlying the odor-related modulation of exercise responses.

## 6 Conclusion

This exploratory study provides proof-of-concept evidence that chocolate-related olfactory stimulation may alter subjective appetite and odor pleasantness while increasing resistance exercise volume during a fasted, single-joint leg extension task. The 90DC condition was characterized by lower hunger and greater fullness, whereas the 60MC condition was characterized mainly by greater odor pleasantness. Both odor conditions increased the total repetitions without clear differences in post-set RPE. However, exploratory mediation analyses did not establish hunger, fullness, or pleasantness as explanatory mechanisms for the performance differences. Overall, these findings suggest that food-related odors may influence the subjective appetite and perceptual context of fasted resistance exercise. However, the proposed appetite-related, affective, and cephalic-phase mechanisms remain preliminary and require direct testing using objective physiological, neuroendocrine, autonomic, and neurophysiological measures.

## Data Availability

The raw data supporting the conclusions of this article will be made available by the authors, without undue reservation.
